# Integrated Transcriptomic and Metabolomic Analyses Reveal the Importance of the Terpenoid, Fatty Acid, and Flavonoid Pathways in Rice Cell Death and Defense

**DOI:** 10.3390/plants14050665

**Published:** 2025-02-21

**Authors:** Pengfei Bai, Yanfang Liu, Laisa Gomes-Dias, Rachel Combs-Giroir, Shaoxing Dai, Naeyeoung Choi, Yun Lin, Matthew Bernier, Emmanuel Hatzakis, Guo-Liang Wang, Joshua J. Blakeslee

**Affiliations:** 1 Department of Plant Pathology, The Ohio State University, Columbus, OH 43210, USA; pengfei.bai1988@gmail.com (P.B.); liuyf528@163.com (Y.L.); choi.1606@buckeyemail.osu.edu (N.C.); 2 Quality Standard and Testing Technology Research Institute, Yunnan Academy of Agricultural Sciences, Kunming 650200, China; 3 Department of Horticulture and Crop Science, The Ohio State University, Columbus, OH 43210, USA; laisa.ufg@gmail.com (L.G.-D.); combs-giroir.1@buckeyemail.osu.edu (R.C.-G.); lin.1418@buckeyemail.osu.edu (Y.L.); 4 Laboratory for the Analysis of Metabolites from Plants (LAMP), The Ohio State University, Columbus, OH 43210, USA; 5 Food Science and Technology Program, Federal University of Tocantins, Palmas 77001, TO, Brazil; 6 Center for Applied Plant Sciences, The Ohio State University, Columbus, OH 43210, USA; 7 Institute of Primate Translational Medicine, Kunming University of Science and Technology, Kunming 650500, China; daisx@lpbr.cn; 8 Campus Chemical Instrumentation Center (CCIC), The Ohio State University, Columbus, OH 43210, USA; bernier.19@osu.edu; 9 Department of Food Science and Technology, The Ohio State University, Columbus, OH 43210, USA; chatzakis.1@osu.edu

**Keywords:** rice blast, cell death, metabolomics, transcriptomics, terpenoid, gibberellins

## Abstract

Lesion mimic mutants provide unique tools to investigate plant–pathogen interactions, often exhibiting hypersensitive responses in the absence of biotic or abiotic stresses. The overexpression of the S-domain receptor-like kinase gene, *SPL11* cell-*d*eath *s*uppressor *2* (*SDS2*), in rice leads to constitutive programmed cell death and enhanced resistance to fungal and bacterial pathogens. However, the mechanisms underlying this broad-spectrum resistance remain unclear. This study integrates transcriptomic and metabolomic analyses of the *SDS2-ACT* mutant to uncover gene expression and metabolic shifts associated with disease resistance. To identify *SDS2-*specific physiological changes related to pathogen resistance, leaf tissues from the *SDS2-ACT* mutant and the Kitkaake WT line were subjected to both transcriptomic and non-targeted metabolic profiling. Transcriptomic analyses identified 1497 differentially expressed genes (DEGs), including up-regulated genes involved in terpenoid and flavonoid biosynthesis, phytohormone signaling, and defense-related pathways (including pathogenesis-related [PR] genes). Metabolomic profiling revealed significant alterations in the accumulation of several compound classes, including putative: terpenoids, phenylpropanoids, phytohormones, fatty acids, and sugars. These changes are likely correlated with the observed cell death and resistance phenotypes in the SDS2-ACT mutant. This study provides an overall landscape of the transcriptomic and metabolomic alterations in a lesion mimic mutant, identifying candidate defense-related genes and metabolites for functional analysis in rice.

## 1. Introduction

Rice (*Oryza sativa* L.) is the staple food crop for half of the world’s population [1], with an increase in production of approximately 40% needed by 2030 to meet the demand [2]. However, rice diseases, particularly rice blast caused by *Magnaporthe oryzae* (syn. *Pyricularia oryzae*), result in significant yield losses of 10–30% [3]. Chemical and biological methods are often ineffective and not environmentally friendly. Host resistance remains the most sustainable and safe approach to combat rice diseases [4]. Using genetic and genomic approaches, significant progress has been made in understanding the molecular basis of rice immunity against pathogens over the last three decades [5,6].

Lesion mimic mutants (LMMs) are valuable for studying plant immunity, as they show spontaneous lesions similar to hypersensitive responses without external stresses [7]. To date, over 100 LMMs have been reported in plants, with about 50 of these LMMs being genetically and/or physiologically characterized [8,9]. Most LMM genes are controlled by single recessive genes encoding a range of proteins functioning in disease resistance, protein degradation, “primary” or “basal metabolism”, gene transcription, signal transduction, phytohormone signaling, and the regulation of cellular energy fluxes. A common feature of LMMs is that the mutations responsible for the lesion formation impact multiple signaling pathways, leading to pleiotropic phenotypes that often result in an HR-like cell death and, in some cases, enhanced resistance to pathogens. Because of the linked nature of the signaling, phytohormone, and metabolic pathways, small alterations in a single LMM gene can result in a “cascade effect”, resulting in large-scale (often at organismal level) changes in the transcriptome, proteome, metabolome, and, ultimately, whole-plant phenotype. Previously completed analyses of the proteomes of LMM lines have indicated that shifts in metabolism at both the cellular and tissue/organ levels may be contributing to disease-resistant phenotypes. For example, many LMM lines exhibited alterations in the levels of proteins or enzymes linked to the metabolism of reactive oxygen species (ROS; indicating alterations to the redox status), basal metabolism, protein synthesis, and/or photosynthesis [10].

Transcriptional profiling of LMMs has also revealed significant up-regulation of genes associated with defense/stress and PCD responses [11,12]. Furthermore, most LMMs also exhibit an up-regulation of genes involved in protein synthesis (i.e., synthesis of ribosomes, biosynthesis of amino acids, and ribosome biogenesis) and a down-regulation of genes involved in photosynthesis. For example, microarray-based transcriptome profiling of the rice *spotted leaf 5* (*spl5*) mutant revealed that the mutation constitutively activates a large number of genes involved in biotic defense responses and ROS metabolism [13]. Interestingly, the expression level of the transcription factor gene *OsWRKY14*, as well as the genes responsible for the biosynthesis of serotonin, anthranilate synthase, indole-3-glycerolphosphate synthase, tryptophan synthase, and tryptophan decarboxylase are significantly up-regulated in the *spl5* mutant [13]. Tryptophan metabolism pathways are important components of the plant immune system. RNA-seq analysis of the rice mutant *early lesion leaf 1* (*ell1*) revealed that genes in the amino acid metabolism and tryptophan biosynthesis pathways are significantly enriched in the differentially expressed genes (DEGs) in the mutant [14]. These results demonstrate that LM mutations affect the expression of many genes that are involved in cell death and defense responses in plants.

Metabolomic techniques enable the identification of biomolecules involved in plant perception, signaling, and adaptive responses to both abiotic and biotic stresses. A recent study of three maize LMM mutants used an integrated transcriptomic and metabolomic approach to identify the populations of genes expressed either commonly across all three lines or specifically within individual lines [12]. Genes involved in PCD, defense responses, and phenylpropanoid and terpenoid biosynthesis were the most commonly up-regulated groups in these three mutants, and, correspondingly, metabolites in the phenylpropanoid, lignin, flavonoid, and terpenoid biosynthesis pathways showed increased accumulation in these LMMs [12].

To date, over 30 LMMs have been identified that are involved in plant immune responses and cell death in rice [9]. The LMM *spotted leaf 11 (spl11)* confers broad-spectrum resistance to *M. oryzae* and *Xanthomona oryzae* pv. *oryzae (Xoo)* [15]. *SPL11* encodes a U-box/armadillo repeat protein conferring E3 ubiquitin ligase activity and plays a negative role in regulating PCD and resistance via ubiquitination-mediated protein degradation. Interactions between SPL11 and the Rho GTPase-activating protein (RhoGAP), SPL11-INTERACTING PROTEIN 6 (SPIN6), negatively regulate Rho GTPase OsRac1, suppressing OsRac1-mediated immune signaling [16]. As an *spl11* cell death suppressor (SDS), the S-domain receptor-like kinase SDS2 positively regulates PCD and immunity through its interaction with SPL11 [17]. Similarly to other LMMs, the *SDS2* activation tagging line (*SDS2-ACT*) exhibits a dwarf phenotype and enhanced disease resistance. Excessive levels of ROS are generated at and surround the lesions induced in leaf tissues by the *SDS2-ACT* mutation. One reason for this is that SDS2 interacts with the receptor-like cytoplasmic kinase OsRLCK118, which phosphorylates the NADPH oxidase OsRbohB to induce an ROS burst. Therefore, the cell death and defense phenotypes in *SDS2-ACT* are potentially the cumulative results of E3 ligase SPL11- and OsRLCK 118/OsRbohB-mediated PCD in which the altered defense signaling cascades (including the production of high levels of ROS) cause a large reprogramming of transcriptome and metabolome in the surrounding tissues.

Here, we present data detailing the major transcriptomic and metabolomic changes observed in the *SDS2-ACT* line compared to the wild-type (WT) Kitaake. We hypothesized that the *SDS2-ACT* mutant exhibits significant alterations in defense-related metabolic and signaling pathways, which contribute to enhanced resistance to fungal and bacterial pathogens. This study aimed to use an integrated transcriptomic and metabolomic approach to identify these pathways. By examining both transcriptomic and metabolomic data sets in parallel, we obtained a global understanding of the LMM phenotype leading to disease resistance. The incorporation of the metabolomic data set allows a better understanding of the “real-time” impact of changes induced in *SDS2-ACT* and the localized biochemical environment to which pathogens are exposed. Our transcriptomic analyses identified 1497 differentially expressed genes (DEGs), including up-regulated genes involved in terpenoid and flavonoid biosynthesis, phytohormone signaling, and defense-related pathways (including pathogenesis-related [PR] genes), suggesting the possible involvement of the DEGs in base carbon metabolism (sugar, amino acid, and glycerophospholipid synthesis), pathogen responses, secondary metabolism (particularly phenylpropanoid biosynthesis), as well as phytohormone metabolism. Our non-targeted metabolomic data set supports the transcriptomic data and indicates that terpenoids (potentially phytotoxic to pathogens), flavonoids (able to quench ROS species), phytohormones (involved in pathogen-responsive signaling cascades and defense responses) are likely involved in disease resistance in *SDS2-ACT*. These results have provided new insights into the molecular basis of a receptor-like kinase-mediated cell death and defense in plants and numerous candidate genes for engineering broad-spectrum resistant plants.

## 2. Results

### 2.1. Transcriptomic Profiling of SDS2-ACT

**Figure 1:**
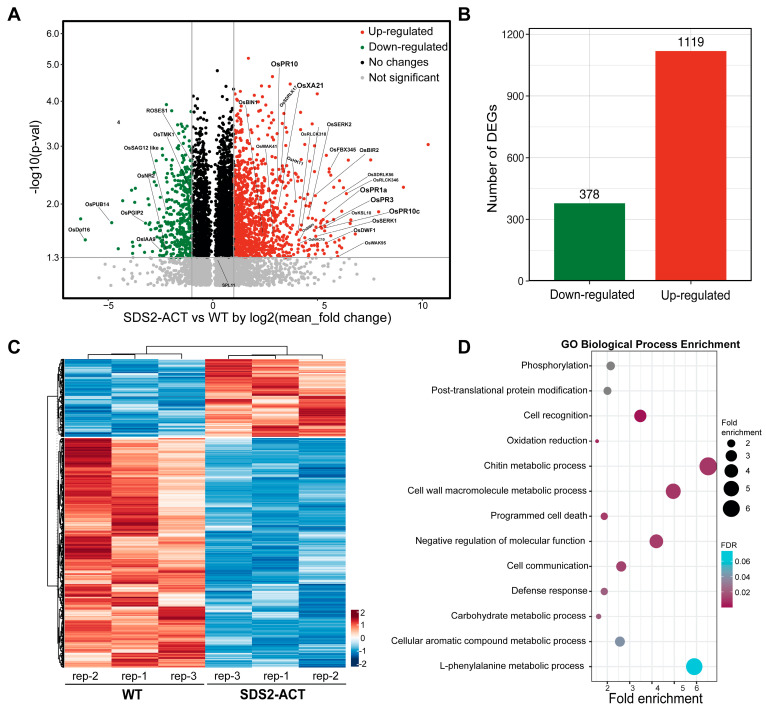
Transcriptomic profiling of rice mutant SDS2-ACT with constitutively activated immunity and cell death. (**A**) Volcano plot of all differentially expressed genes (DEGs) in SDS2-ACT compared to wild-type (WT) rice. The y-axis represents the significance level (-log10(*p*-value)), and the x-axis shows the log2(fold change). Green dots indicate down-regulated genes, and red dots indicate up-regulated genes with significant fold change >2 and *p* < 0.05. (**B**) Bar chart showing the number of DEGs. A total of 378 genes were down-regulated, and 1119 genes were up-regulated in SDS2-ACT compared to WT, indicating a significant shift in gene expression patterns. (**C**) Heatmap representation of DEGs in SDS2-ACT vs. WT rice. The heatmap is a result of one-dimensional hierarchical clustering of the 378 down-regulated and 1119 up-regulated DEGs. Columns represent three independent biological replicates. (**D**) Gene Ontology (GO) analysis of the 378 down-regulated and 1119 up-regulated DEGs highlights the enrichment of defense and cell death-related pathways. The dot plot shows the top enriched GO terms in biological processes, with the size of the dots representing fold enrichment and color indicating the false discovery rate (FDR).

To investigate the molecular mechanisms underlying SDS2-regulated defense and cell death, we conducted transcriptomic profiling of the *SDS2-ACT* rice mutant. The transcriptomic analysis identified 1497 DEGs with at least a 2-fold change and a *p*-value of less than 0.05 when comparing *SDS2-ACT* to WT (Figure 1A, Table S1). Of these DEGs, 1119 genes were significantly up-regulated, while 378 genes were down-regulated (Figure 1B,C). The heatmap in Figure 1C indicates distinct expression patterns, suggesting significant transcriptional reprogramming in the *SDS2-ACT* mutant. Gene Ontology (GO) analysis was performed on the 1497 DEGs to identify biological processes enriched in *SDS2-ACT* vs. WT or vice versa (Figure 1D). GO analyses indicated that several pathways implicated in plant defense responses and cell death were enriched in SDS2-ACT vs. WT (Figure 1D).

In the area of plant defense responses, genes involved in chitin metabolic processes, cell wall macromolecule metabolic processes, programmed cell death, and general “defense responses” were among the most enriched. The enrichment of genes involved in programmed cell death aligns well with the cell death phenotype exhibited by *SDS2-ACT* mutants [17]. In addition to pathways directly associated with defense responses and cell death, GO analyses also identified several pathways showing enrichment in *SDS2-ACT*, which could be either directly or indirectly involved in plant stress responses and/or pathogen defense. These pathways included “negative regulation of cell function”, L-phenylalanine metabolic processes, aromatic compound metabolic processes, post-translational protein modification, and phosphorylation (Figure 1D).

We next scanned our transcriptomic data to identify specific pathogen-response (PR) genes showing increased expression in *SDS2-ACT* vs. WT (Figure 1C, Figures S1 and S2A). In these analyses, we identified several PR genes, including *OsPR1a*, *OsPR1-12*, *OsPR2*, *OsPR3*, *OsPR4*, *OsPR5*, *OsPR10*, *OsPR10c*, and *OsPR6*. *OsPR1a* and *OsPR1-12* are well-known markers of systemic acquired resistance (SAR), playing a vital role in conferring broad-spectrum disease resistance [18]. The significant up-regulation of these genes in the *SDS2-ACT* mutant suggests an enhanced SAR response, potentially contributing to increased resistance against a wide range of pathogens. *OsPR3* and *OsPR4* encode chitinases, which degrade chitin in fungal cell walls, and the increased expression of these genes could potentially inhibit fungal growth [19]. In addition to *PR* genes, our transcriptomic analyses identified several DEGs in metabolic pathways linked to plant defense/stress responses, and pathogen responses showed increased expression in *SDS2-ACT* mutants; and increased expression in a sub-set of these genes was confirmed via quantitative real-time PCR analyses (Figure S2A,B). Among these DEGs are genes involved in auxin responses (*OsIAA9*) and diterpenoid biosynthesis (*OsKS4*, *OsKS10*) (Figure S2A,B).

### 2.2. KEGG Pathway Enrichment and Network Analysis

**Figure 2:**
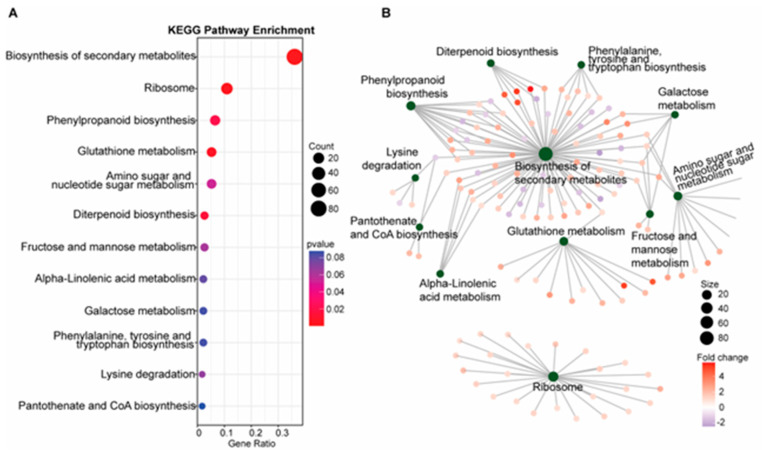
KEGG pathway analysis and gene-concept network visualization (**A**). (**A**) KEGG pathway enrichment analysis of differentially expressed genes (DEGs), highlighting potentially enriched metabolic pathways. The bar chart shows the gene ratio for each significantly enriched pathway, including the biosynthesis of secondary metabolites, phenylpropanoid biosynthesis, and glutathione metabolism. The size of the dots represents the gene count, and the color indicates the *p*-value, with red indicating higher significance. (**B**) Gene-concept network. The network diagram visualizes the connections between multiple biological categories and the specific DEGs involved in those pathways. Nodes represent KEGG pathways, and edges represent DEGs connected to these pathways. Node size indicates the number of genes involved, and the color of the edges represents the fold change in gene expression.

To further define specific genetic and metabolic pathways potentially involved in blast resistance in the *SDS2-ACT* mutant, we completed KEGG pathway enrichment and gene-concept network analyses, as illustrated in Figure 2.

Unsurprisingly, the most significantly enriched pathway was “biosynthesis of secondary metabolites”, a pathway that encompasses several metabolic pathways previously implicated in fungal resistance, including phytoalexins, flavonoids, and polyterpenoid compounds. Another significantly enriched pathway was phenylpropanoid biosynthesis, which previous mGWAS analyses have implicated in blast resistance [20] and which is responsible for the production of flavonoids, anthocyanins, and other phenolic compounds involved in cell wall synthesis, including lignins. Consistent with the increased expression of DEGs involved in phenylpropanoid metabolism, our KEGG pathway analyses also showed an increased expression of genes involved in phenylalanine (a key precursor of the phenylpropanoid synthesis pathway), tyrosine, and tryptophan biosynthesis in *SDS2-ACT*. KEGG pathway analyses also indicated an enrichment of glutathione metabolism in *SDS2-ACT*. As glutathione is an antioxidant that protects cells from oxidative damage, the enrichment of this pathway could be associated with the constitutive ROS phenotype displayed by *SDS2-ACT* mutants. Finally, *SDS2-ACT* mutants displayed an enrichment of genes involved in the synthesis of diterpenoids. In rice, diterpenoids such as momilactones, oryzalexins, and phytocassanes have been identified as key phytoalexins that contribute to resistance against a wide range of pathogens, including fungi and bacteria [21]. Further, diterpenoids serve as the precursors of gibberellin, a phytohormone involved in both plant development and pathogen responses.

### 2.3. Comparative Analysis of DEGs in SDS2-ACT Mutant and Pathogen-Responsive Genes

**Figure 3:**
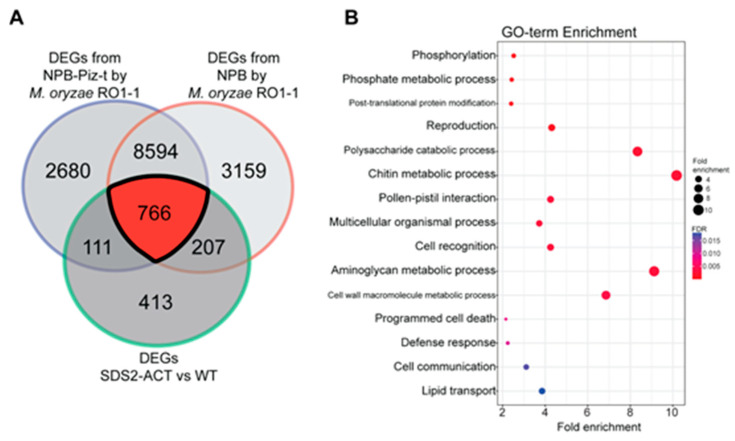
Comparative analysis of DEGs from SDS2-ACT vs. WT with DEGs from NPB and NPB-Piz-t rice groups sprayed with *Magnaporthe oryzae* strain *RO1-1* (**A**). (**A**) The Venn diagram highlights the overlapping and unique DEGs between the SDS2-ACT vs. WT comparison and the compatible/incompatible interaction from NPB and NPB-Piz-t rice infected with *M. oryzae* strain *RO1-1*. There are 766 common DEGs identified across these conditions. (**B**). The 766 common DEGs from the Venn diagram were subjected to GO term enrichment analysis. The analysis identified several significantly enriched defense-related GO terms, emphasizing the role of these DEGs in the plant’s defense mechanisms. The enriched GO terms include processes related to immune response, signal transduction, and cellular defense mechanisms, highlighting their potential involvement in the enhanced defense response in the SDS2-ACT mutant.

To further investigate the DEGs identified in the *SDS2-ACT* transcriptomic analysis, we compared these genes with DEGs identified in a parallel transcriptomic study employing the blast-resistant *Piz-t* line and its associated wild-type susceptible line Nipponbare, both inoculated with the fungal pathogen *M. oryzae* RO1-1 [22]. DEGs were identified across various time points (0, 24, 48, 96, and 120 h) post-inoculation, with 0 h post-infection used as the reference (i.e., “control”) time point. The Venn diagram in Figure 3A shows the overlap between DEGs from the *SDS2-ACT* mutant and the pathogen-responsive DEGs from both the resistant and susceptible groups. Notably, there was a significant overlap of 766 genes between the DEGs of the *SDS2-ACT* mutant and those from the *Piz-t* resistant and susceptible lines. The substantial overlap with pathogen-responsive genes from resistant and susceptible lines confirms the involvement of these DEGs in key defense mechanisms, suggesting that the constitutive activation of the RLK in the *SDS2-ACT* mutant primes the plant for enhanced defense readiness.

GO term enrichment analysis of the overlapping DEGs, presented in Figure 3B, indicates significant enrichment in biological processes related to defense response, programmed cell death, and various cellular signaling and metabolic processes. Key enriched processes include phosphorylation and post-translational protein modification in the category of regulatory processes, and both chitin and cell wall macromolecule metabolism in the area of miscellaneous metabolic processes. These findings reinforce the hypothesis that the *SDS2-ACT* mutant activates multiple defense responses, integrating multiple signaling pathways and metabolic processes.

### 2.4. Metabolomic Profiling of SDS2-ACT Mutant

**Figure 4:**
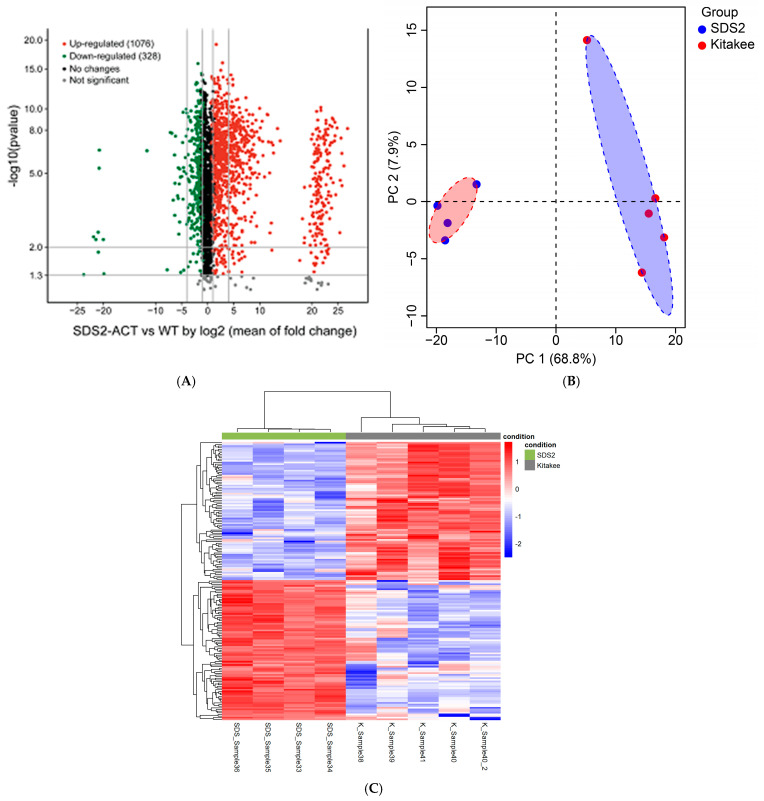
Metabolomic profiling of rice mutant SDS2-ACT compared to WT. (**A**) Volcano plot representing differential metabolite levels between the SDS2-ACT mutant and WT plants. Metabolites with significant up-regulation are depicted in green, while significantly down-regulated metabolites are shown in red. Black dots indicate metabolites with no significant changes. (**B**) Principal component analysis (PCA) plot demonstrating the separation of metabolomic profiles between the SDS2-ACT mutants (red) and WT plants (blue). (**C**) Heatmap illustrating the concentration patterns of differentially abundant metabolites in the SDS2-ACT mutants compared to WT plants. Rows represent individual metabolites, and columns represent biological replicates.

Overview: In parallel with the transcriptomic profiling experiments detailed above, we conducted non-targeted, broad-spectrum/“pathway-level” metabolic profiling studies of *SDS2-ACT* vs. WT using the leaves of 5-week-old seedlings and LC-qTOF-MS analyses. Feature identification was performed using a custom algorithm to screen features against the KEGG, Plant Metabolic Network (PMN), and Chemspider databases. A volcano plot of the metabolomic data (Figure 4A) illustrates the differences in metabolite accumulation between *SDS2-ACT* and WT. Interestingly, the features (individual metabolites) with increased accumulation in *SDS2-ACT* (red dots) showed, on average, a greater range in log2-fold change and, in many cases, greater log2-fold changes (in terms of degree of shift in accumulation) than metabolites with decreased accumulation in *SDS2-ACT* (green dots). A total of 1404 metabolites showed significant changes in *SDS2-ACT* vs. WT, with 1076 metabolites up-regulated and 328 down-regulated in the *SDS2-ACT* mutant (Figure 4A, Table S2). Principal component analysis (PCA) of the metabolomic data showed a clear difference between the *SDS2-ACT* and WT lines (Figure 4B). The differences between the *SDS2-ACT* and WT metabolome are further demonstrated when the data are used to generate a clustered heatmap (Figure 4C). The top 200 up- and down-regulated compounds in *SDS2-ACT* were selected based on log-fold change in the normalized peak area of these features compared to WT, and algorithmically assigned putative compound identities were manually curated by cross-checking across the databases listed above (Tables S2 and S4; while these tables each contain 200 metabolites, they may contain more than 200 entries due to some metabolites/features having more than one possible identity). While the resolution of the metabolomic analyses conducted did not allow the definitive confirmation of species identity (which would require additional, targeted mass spectrometric analyses, and likely the application of nuclear magnetic resonance [NMR]), analyses of mass spectrometric features and the assignment of putative compound identities did allow the identification of several pathways, showing altered metabolite accumulation in the *SDS2-ACT* mutant. Based on these data, we identified the metabolic pathways impacted the most in *SDS2-ACT* as the following: terpenoid and sterol metabolism (70 putative metabolites; 55 increased, 15 decreased); flavonoid metabolism (45 putative metabolites; 11 increased, 34 decreased); phytohormone metabolism (22 putative metabolites; 15 increased, 7 decreased); fatty acid metabolism (18 putative metabolites; 14 increased, 4 decreased); amino acid metabolism (14 putative metabolites; 7 increased, 7 decreased); phenylpropanoid metabolism (13 putative metabolites; 9 increased, 4 decreased); alkaloid metabolism (12 putative metabolites; 11 increased, 1 decreased); and carbohydrate metabolism (11 putative metabolites; 3 increased, 8 decreased) (Table S3, up-regulated, and Table S5, down-regulated). Additional metabolic pathways identified as being up-regulated in SDS2-ACT included polyamine and saponin biosynthesis, while additional pathways identified as being down-regulated in these lines included anthocyanin, riboflavin, and glutathione metabolism. Observed changes in these metabolic pathways are described in more detail below.

Terpenoids and sterols: Terpenoids and sterols were the most well-represented metabolite classes in the list of putative features exhibiting altered accumulation in *SDS2-ACT* lines. Sterols and sterol derivatives appeared to be most impacted by *SDS2* activation, with twenty putative sterols being up-regulated and four sterols being down-regulated in the line. Interestingly, one of the sterols most up-regulated in *SDS2-ACT*, and the ninth-highest increased compound in the line, was tentatively identified as castasterone 23-o-α-d-glucoside, which functions as an end-product of brassinosteroid metabolism in rice. While the resolution of the qTOF-MS performed in this study did not allow the resolution of many sterol derivatives beyond compound class, the data did allow the determination that several putative sterol metabolites present were likely 3-hydroxy-steroids.

In alignment with the observation that levels of sterols and sterol derivatives were the largest class of terpenoid molecules exhibiting altered accumulation in *SDS2-ACT*, the second largest class of terpenoid molecules exhibiting changes in accumulation was the combined group of sesqui- and triterpenoid molecules (grouped together in our analyses as two 15-carbon sesquiterpenoid farnesyl pyrophosphate molecules joined together to make squalene, the 30-carbon precursor of all triterpenoid molecules). LC-MS analyses indicated that 21 putative sesqui- and triterpenoids showed increased accumulation in *SDS2-ACT*, while one sesquiterpenoid species was decreased in these plants. The putative sesqui- and triterpenoids of particular interest which were found at higher levels in *SDS2-ACT* included the following: convallagenin A (a triterpenoid precursor of convallagenin saponin), kikkanol A (a germacrene sesquiterpenoid proposed to function as an anti-bacterial and anti-fungal compound [23], and (−)-β-Caryophyllene epoxide (a cyclic sesquiterpenoid linked to antimicrobial activity).

Finally, several tentatively identified mono- and diterpenoids exhibited altered levels of accumulation in *SDS2-ACT*. Most of the mono- and diterpenoids putatively identified showed increased levels of accumulation in *SDS2-ACT*. Compounds potentially contributing to resistance in these categories included the following: (−)-abietic acid (a cyclic diterpenoid linked to microbial resistance in coniferous species), oryzalexin A (a diterpenoid phytoalexin found in several rice species) [24], an iridoid glycoside with structural similarity to harpagoside, 6β,7β-Dihydroxykaurenoic acid (a diterpenoid precursor of gibberellins), several beta-carotene precursors, and a putative terpenoid alkaloid with strong spectral similarity to taxine A and the indolic alkaloid vobtusine, which was the most highly accumulated putative metabolite in *SDS2-ACT*.

Flavonoids: In addition to terpenoids and sterols, several molecular species tentatively identified as flavonoids exhibited large shifts in accumulation in *SDS2-ACT*. However, while most of the putative terpenoids and sterols putatively identified in our screen showed increased accumulation in *SDS2-ACT*, the majority of the flavonoid compounds putatively identified in our study instead showed decreased accumulation in this line compared to WT (34 compounds with decreased accumulation, 11 with increased accumulation). Flavonoid species showing decreased accumulation in *SDS2-ACT* were in large part tentatively identified as flavonols and flavones, as well as derivatives (primarily glycosides) of these species. Flavonols and flavones showing decreased accumulation in *SDS2-ACT* included the following: quercetin 3-o-rhamnoside-7-o-glucoside, kaempferol-3,7-bis-o-α-l-rhamnoside, putative kaempferol derivatives (primarily glycosides), isorhamnetin 3-glucoside, and several potential di- and tri-hydroxyflavanones and hydroxyflavones. The flavonoid compounds showing increased accumulation in *SDS2-ACT* were flavonols linked to pathogen defense, including the following: kurarinol (the 5th most over-accumulated compound in *SDS2-ACT*) and kuraridinol (both kurarinol and kuraridinol are tyrosinase inhibitors [25]), flavonol 3-o-(6-o-malonyl-β-d-glucoside), and (−)-(*S*)-sakuranetin (a rice phytoalexin which had been demonstrated to prevent the germination of *P. oryzae* [26]).

Phytohormones: Unsurprisingly, given the pleiotropic developmental phenotypes in *SDS2-ACT*, the third-largest category of compounds showing altered levels of accumulation in the line were phytohormones and phytohormone derivatives. While the extraction protocol used in this study was not optimized for the extraction and quantification of phytohormones, which usually requires multiple purification and concentration steps prior to the application of targeted, triple quadrupole LC-MS/MS, our broad-spectrum profiling approach was able to identify several metabolite precursors and degradation products (showing both increased and decreased accumulation), allowing us to determine which phytohormone pathways are involved with *SDS2-ACT* phenotypes and disease resistance. The hormone pathway apparently most impacted in *SDS2-ACT* was cytokinin synthesis. The *SDS2-ACT* line showed large increases in the levels of N-glycosylzeatin present in leaves, and the levels of dihydrozeatin riboside and enadenine were also increased in the tissue. Interestingly, the levels of cis-zeatin riboside monophosphate and trans-zeatin were decreased. Jasmonic acid levels were also impacted, with methyl jasmonate, (−)-Jasmonoyl-l-isoleucine, 6-jasmone, and 3,6-nondienal all showing increased accumulation in *SDS2-ACT*. Given the general increase in terpenoid metabolites in *SDS2-ACT* detailed above, it was not surprising that several terpenoid-based phytohormone metabolites were also increased in the line. For example, as noted above, the levels of the brassinolide castasterone were increased in *SDS2-ACT*, as was an additional unidentified brassinolide metabolite, gibberellic acid A53, and a breakdown product of GA-A34. Not all terpenoid phytohormone metabolites observed in *SDS2-ACT* increased; however, the levels of gibberellin A4 and phaseic/phaseolic acid (an abscisic acid catabolism product with diterpenoid precursors) were decreased in this line. Finally, while the extraction procedure used in this study made it unlikely that auxins or auxinic compounds, noted for their labile nature and susceptibility to oxidation during compound extractions, would be easily quantifiable in our assays, the LC-MS results did indicate that the levels of phenylacetic acid (PAA), an auxinic compound increasingly linked to auxin metabolism and stress response pathways [27,28,29,30], was significantly increased in *SDS2-ACT*.

Fatty acids: Given the overproduction of ROS associated with LMMs in general, and *SDS2-ACT* in particular, it was not surprising that our metabolomic analyses identified increased levels of several oxidized and/or hydroxylated lipids in these plants. Most of these lipids fell into the C16–C18 range, indicating that they are likely derived from membrane glycrophospholipid metabolism. Putative lipids showing increased accumulation in *SDS2-ACT* included the following: 16-hydroxyhexadecanoate, 12-hydroxyheptadecatrienoic acid, 2-methoxy-5Z-hexadecenoic acid, (*R*)-2-hydroxystearic acid, 10-nitro-9Z,12Z-octadecadienoic acid, and linolenic acid. Additionally, several long-chain lipids involved in wax synthesis were increased in *SDS2-ACT*. Few lipids showed decreased accumulation in *SDS2-ACT*, and most of these were short-chain fatty acids or short-chain fatty acid diols.

Phenylpropanoids: As noted above, flavonoid metabolism appears to be altered in *SDS2-ACT*. In addition to the decreases in many flavonoid species detailed above, our LC-MS data also showed that *SDS2-ACT* plants also exhibited changes in the levels of several phenylpropanoid compounds either upstream of flavonoids or in parallel metabolic pathways. *SDS2-ACT* showed increased accumulations of several putative compounds previously associated with either stress or defense responses, including the following: 3,4-hydroxybenzoate, coumarin, cinnamyl alcohol, and dihydroconiferol alcohol. Somewhat surprisingly, however, several putative phenylpropanoid compounds linked to defense and/or pathogen responses showed decreased accumulation in *SDS2-ACT*. For example, *cis-p-*coumarate, an unknown coumarin, the lignan glycoside arctiin, and (−)-deoxypodophyllotoxin were all found at levels lower than WT in *SDS2-ACT*.

Alkaloids: While not particularly abundant in most rice lines, our metabolic screening of *SDS2-ACT* vs. WT leaf tissue did identify several putative alkaloids as being accumulated to higher levels in *SDS2-ACT*. However, due to the structural complexity of alkaloids, the confirmation of these species’ identities will require the application of higher-resolution mass spectrometric and NMR techniques.

Other compounds of interest: As described above, small groups of 1–5 metabolites from several other compound classes, including amino acids, sugars, riboflavins, and other potential defense compounds exhibited differential accumulation between *SDS2-ACT* and WT plants. Compounds exhibiting increased accumulation in *SDS2-ACT* include the following: serotonin (an indolic signaling compound), porphyrin (chlorophyll biosynthesis), and 4-hydroxybenzaldehyde (a phenolic compound). Metabolites related to pathogen responses showing decreased accumulation in *SDS2* included the following: glutathione, dopaxanthin, 18:2-18:3 digalactosyl diacylglycerol (DGDG; a plastidic membrane lipid), 3,4-dihydroxy-5-methoxybenzoic acid (a phenolic compound), calcium oxalate, 4-sinapoylbutylglucosinolate (a glucosinolate), and both methylcytidine and methyladenine.

### 2.5. Transcriptomic and Metabolomic KEGG Pathway Analysis of SDS2-ACT vs. Kitaake

Overview: To further identify pathways involved in pathogen resistance in *SDS2-ACT* mutants, we performed a combined analyses integrating both metabolomic and transcriptomic data sets. In these analyses, we used the KEGG pathway to identify metabolic pathways with outputs altered in *SDS2-ACT* compared to wild type. In support of the transcriptomic and metabolomic data detailed above, our combined KEGG pathway analyses highlighted terpenoid metabolism as being altered in *SDS2-ACT*. Specifically, our combined transcriptomic and metabolomic analyses identified diterpenoid and carotenoid metabolism and downstream phytohormone metabolic pathways (momilactone biosynthesis and gibberellin biosynthesis for diterpenoids, and abscisic acid for carotenoids) as targets of the *SDS2-ACT* mutation resulting in increased pathogen resistance.

**Figure 5:**
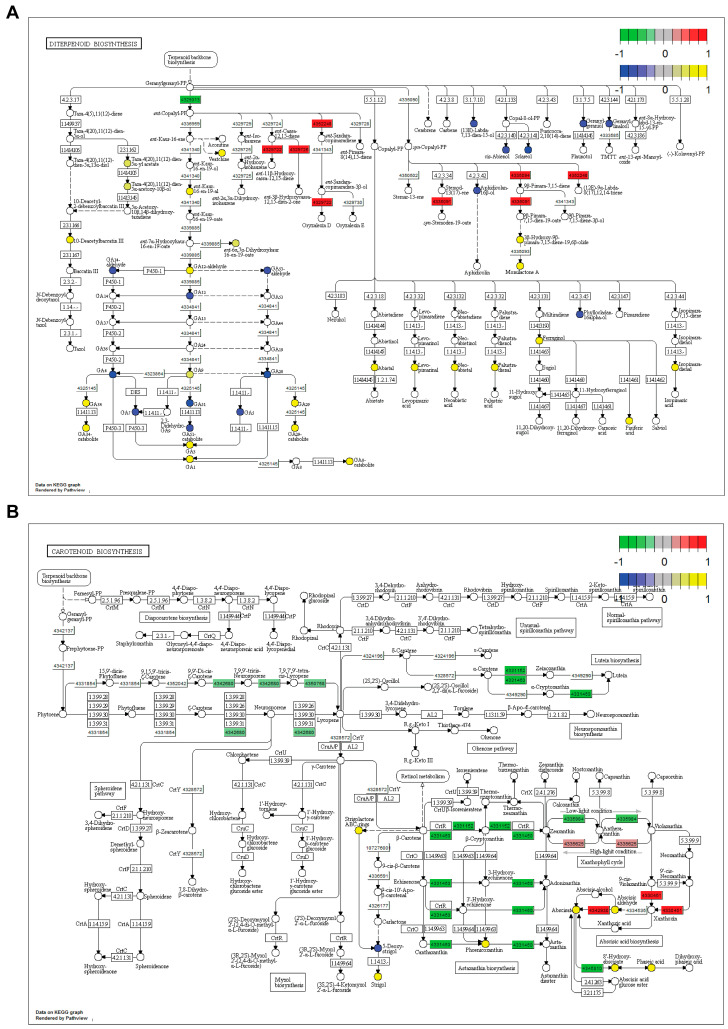
Combined transcriptomic and metabolomic pathway analysis in SDS2-ACT. Kyoto Encyclopedia of Genes and Genomes (KEGG) pathway analysis of transcriptomic and metabolomic data in SDS2-ACT vs. WT. (**A**) Pathway analysis showed altered levels of expression of genes involved in diterpene biosynthesis in SDS2-ACT vs. WT. (**B**) Pathway analysis showed altered expression of genes involved in carotenoid metabolites in SDS2-ACT vs. WT. A, B. Red boxes indicate up-regulated genes, while green boxes indicate down-regulated genes. Circles indicate specific metabolites. Yellow circles indicate metabolites with increased accumulation in SDS2-ACT, as determined by LC-MS analyses; blue circles indicate metabolites with decreased accumulation in SDS2-ACT vs. WT.

Diterpenoid metabolism: KEGG pathway analyses showed that several branches of diterpenoid metabolism were altered in *SDS2-ACT* lines compared to the Kitaake wild type (Figure 5A). Our metabolomic analyses identified oryzalexin A as showing increased accumulation in *SDS2-ACT*. In agreement with this, our pathway analysis showed increased activity of the oryzalexin synthesis pathway in *SDS2-ACT*, with two genes encoding the enzymes catalyzing the two penultimate steps of oryzalexin D synthesis (ent-sendaracopimaradiene/labdatriene synthase [43652248]; oryzalexin D synthase [4329722]) increased in this mutant (Figure 5A). Further, our pathway analyses identified the momilactone A synthesis pathway as being up-regulated in *SDS2-ACT*, with two genes (syn-pimara-7,15-diene synthase [4335094]; 9-beta-pimara-7,15-diene oxidase [4335091]) and two metabolites (3-beta-hydroxy-9-beta-pimara-7,15-diene-19,6-beta, olide; momilactone A) showing increased expression and accumulation, respectively, in the mutant line compared to Kitaake. This is particularly significant, as momilactones A and B have been demonstrated to function as anti-fungal compounds contributing to pathogen resistance in rice [31].

In addition to anti-fungal compounds, our KEGG pathway analyses expanded on our transcriptomic and metabolomic analyses and further supported a role for gibberellins in the pathogen-resistance phenotype of *SDS2-ACT* (Figure 5A). Expanding on the transcriptomic and metabolomic results detailed above, our pathway analyses revealed the impact of the *SDS2-ACT* mutation on gibberellin metabolism as complex. While some GAs (GA7, GA51, GA7, GA5, and GA20) and GA metabolites (GA14-aldehyde, GA53-aldehyde, and GA51-catabolite) and one gene involved in GA synthesis (*ent-*copalyl diphosphate synthase [4320913]) showed decreased levels in *SDS2-ACT*; multiple other GAs (GA9, GA34, GA3, GA1, and GA29), GA metabolites (GA34-catabolite, GA12-aldehyde, GA29-catabolite, and GA8-catabolite), and GA precursors (*ent-*kaur-16-en-19-al) showed increased accumulation in this mutant.

Finally, our pathway analyses indicated that several abietadiene-related terpenoid compounds showed altered accumulation in *SDS2-ACT* mutants. Most of these compounds (for example, abietal, levo-pimarinal, pisiferic acid) showed increased accumulation in *SDS2-ACT*, and at least one of these compounds (hyllocladane-16-alpha-ol) showed increased accumulation in this mutant.

Carotenoid metabolism: In addition to diterpenoid metabolism, our KEGG pathway analyses integrating both transcriptomic and metabolomic data indicated that *SDS2-ACT2* lines also exhibited alterations in carotenoid metabolism (Figure 5B). Interestingly, one of the primary carotenoid pathway clusters impacted by the *SDS2-ACT* mutation is the pathway group containing lycopene and lutein biosynthesis. The expression levels of several genes encoding enzymes catalyzing key steps in these pathways, including beta-carotene-3-hydroxylase (4331460), prolycopene isomerase (4350758), zeta-carotene desaturase (4342680), and beta-ring hydroxylase (4331152), were down-regulated in the *SDS2-ACT* mutant (Figure 5B). Additionally, several genes encoding enzymes catalyzed the downstream metabolism of beta-carotene (beta-ring hydroxylase [4331152] and, as noted above, beta-carotene-3-hydroxylase [4331460]). These data are in agreement with our metabolomic data, which showed an increase in beta-carotene levels in *SDS2-ACT*, a piece of data that would correlate well with the down-regulation of pathways using beta-carotene as a metabolic input. Interestingly, despite the down-regulation of several metabolic pathways downstream from beta-carotene, *SDS2-ACT* mutants showed an increased accumulation of one of the metabolites of these pathways (phoenicoxanthin) (Figure 5B).

In addition to lycopenes, luteins, and beta-carotenes, and in agreement with our metabolomic-focused analyses, *SDS2-ACT* mutants showed alterations to abscisic acid synthesis pathways (Figure 5B), while several genes encoding enzymes involved with ABA synthesis (violaxanthin de-epoxidase [4335625], 9-cis-epoxycarotenoid dioxygenase [4330451], and abscisic aldehyde oxidase [4342930]) were up-regulated in *SDS2-ACT* lines. Correspondingly, metabolites produced by these enzymes, including both abscisic aldehyde and abscisic acid, were also increased in *SDS2-ACT* mutants compared to Kitaake WT. Our pathway analyses also indicated that the ABA metabolites 8′-hydroxyabscisic acid and phaseic acid (a compound also found to show increased accumulation in our metabolomics analyses) were increased in *SDS2-ACT*, an interesting result as one of the enzymes regulating the synthesis of these metabolites ((+)-ABA-8′-hydroxylase) showed decreased expression in this mutant.

## 3. Discussion

The combination of transcriptomic and metabolomic analyses conducted in this study allowed the identification of several metabolic pathways potentially involved in disease resistance in *SDS2-ACT*. These findings provide insights into the links between transcriptional and metabolic reprogramming involved in plant immunity. Both transcriptomic and metabolomic data indicated significant alterations in terpenoid/sterol, phenylpropanoid (particularly anthocyanin and flavonoid), phytohormone, fatty acid, transporters, and carbohydrate metabolism in *SDS2-ACT* may play a role in fungal pathogen defense.

The transcriptomic analysis of the SDS2-ACT mutant identified 1497 differentially expressed genes (DEGs). Notably, several pathogenesis-related (PR) genes, including *PR1*, *PR3*, *PR4*, *PR5*, and *PR10*, were significantly up-regulated in the SDS2-ACT mutant. *PR* genes are crucial components of the plant immune system, often being induced during systemic acquired resistance (SAR). They play roles in reinforcing cell walls, degrading pathogen cell walls, and signaling for further defense responses. The up-regulation of these genes suggests an enhanced state of defense readiness in the *SDS2-ACT* mutant, reflecting a primed immune response often associated with SAR. This highlights the activation of receptor kinase-mediated defense mechanisms that may provide broad-spectrum protection against fungal and bacterial pathogens. The transcriptomic results also highlighted the significant up-regulation of genes involved in the biosynthesis of secondary metabolites, such as terpenoids and flavonoids, which are known to play roles in defense mechanisms. The increased expression of genes involved in the synthesis of diterpenoid phytoalexins, such as *CPS4*, *OsKSL10*, *OsKS4*, *OsKS8*, *OsCYP99A2*, and *OsCYP99A3*, suggests the activation of pathways that produce diterpenoid antimicrobial compounds.

Further, transcriptomic, metabolomic, and KEGG pathway analysis data sets all indicated a general up-regulation of terpenoid metabolism in *SDS2-ACT* plants. Examining the gene expression and metabolite accumulation data suggests that carbon flux into terpenoid synthesis is up-regulated and targeted into specific branches of terpenoid synthesis. One of the largest classes of compounds showing increased accumulation in *SDS2-ACT* was the combined group of 15-carbon sesquiterpenoids and 30-carbon triterpenoids (dimers of the 15-carbon sesquiterpene precursor farnesene). The significant accumulation of multiple putative sesquiterpenoid compounds, such as kikkanol and β-caryophyllene epoxide, strongly implicates terpenoid metabolism in the defense response of SDS2-ACT. These compounds are well documented for their antimicrobial activities, suggesting their important roles in limiting pathogen growth and spread. Consistent with the observed increase in sesquiterpenoid accumulation, *SDS2-ACT* also showed increases in the levels of several triterpenoids, particularly sterols. Mass spectrometric analyses indicated that *SDS2-ACT* accumulated increased levels of several putative 3-hydroxy sterols, as well as the brassinolide end-product castasterone 23-o-α-d-glucoside. The transcriptomic data related to sterol metabolism are more complicated, with genes involved in sterol synthesis both up- and down-regulated. Interestingly, however, several genes involved in the synthesis of potential sterol precursors (phosphomevalonic acid phosphate) are up-regulated in *SDS2-ACT*. Alterations in sterol profiles in SDS2-ACT may provide resistance via multiple mechanisms, including the modulation of membrane fluidity and structure. Furthermore, the increased accumulation of castasterone, a brassinolide end-product, aligns with previous studies that link brassinosteroids to enhanced disease resistance to the pathogen black shank (*Phytophthora nicotianae*) and the mycotoxin deoxynivalenol (vomitoxin) in tobacco and Arabidopsis, respectively [32,33]. As castasterone is a likely metabolic end-point for brassinolide synthesis in rice, the accumulation of castasterone 23-o-α-d-glucoside in *SDS2-ACT* likely indicates increased brassinolide synthesis, which may contribute to pathogen resistance [34]. Higher levels of sterols may also contribute to disease resistance by altering membrane structure and fluidity, potentially inhibiting the penetrance of pathogens. Sterols are essential components of detergent-resistant membranes (DRMs or “lipid rafts”), which exhibit increased membrane packing (localized accumulation of membrane phospholipids) and decreased membrane permeability. Finally, it should be noted that the assignment of identities to putative sterols identified in this study was complicated by the tendency of sterols and sterol derivatives to generate similar spectra in MS analyses. Future work incorporating both targeted LC-MS/MS and nuclear magnetic resonance (NMR) analyses will be useful in providing increased resolution and structural information on the sterols showing increased accumulation in *SDS2-ACT*.

Our transcriptomic and metabolomic analyses readily identified both sesquiterpenes and sterols as being altered in *SDS2-ACT*. Our KEGG pathway analyses, which combined both metabolomic and transcriptomic data sets, indicated that diterpenoid and carotenoid metabolic pathways were also altered in *SDS2-ACT*. The up-regulation of oryzalexin synthesis, as indicated by KEGG pathway analysis, is a particularly intriguing finding, because oryzalexins are well known for their potent antimicrobial activity. The accumulation of oryzalexin D in *SDS2-ACT* further supports the role of diterpenoid phytoalexins as critical components of rice immune responses. Oryzalexins were not the only putative anti-fungal compounds identified in our pathway analyses. These studies also indicated that *SDS2-ACT* lines accumulated increased levels of the anti-fungal compound momilactone A. Finally, our pathway analyses indicated that *SDS2-ACT* lines showed a decreased accumulation of the strigolactone metabolite 5′-deoxystrigol (Figure 5B). While it is well known that strigolactones function in mediating plant–fungal interactions, particularly with arbuscular fungi, the impact of the decrease in 5′-deoxystrigol levels in *SDS2-ACT* is unclear, particularly as genes encoding enzymes contributing to the synthesis of strigol and strigolactone rings appear to be increased in this mutant. Because of the structural complexity of strigolactone family members, further study incorporating both targeted LC-MS/MS and NMR approaches is needed to elucidate the specific impacts of the *SDS2-ACT* mutation on the accumulation of specific strigolactone species.

In addition to terpenoid/carotenoid/sterol metabolism, our combined transcriptomic and metabolomic analyses also highlighted the role of altered phenylpropanoid metabolism in generating *SDS2-ACT* phenotypes. Both genetic and metabolomic data sets support the hypothesis that the *SDS2* activation increases carbon flux into the shikimic acid pathway and the enhanced synthesis of aromatic amino acids (tyrosine, phenylalanine, and tryptophan) and downstream metabolic processes. Additionally, transcriptomic analyses indicated an increased expression of phenylalanine ammonium lyase, the rate-limiting step of phenylpropanoid synthesis downstream of the shikimic acid pathway. Increased carbon flux into phenolic metabolism is further supported by the increased accumulation of 3,4-hydroxybenzoate, coumarin, cinnamyl alcohol, and dihydroconiferol alcohol observed in *SDS2-ACT*. These metabolites have previously been linked to disease resistance and may contribute to the disease resistance in *SDS2-ACT*.

In addition to alterations to base phenolic/phenylpropanoid metabolism likely contributing to broad-spectrum resistance in *SDS2-ACT*, these plants also exhibit shifts in more complex downstream phenylpropanoid pathways, particularly carotenoid and flavonoid metabolisms that may also contribute to disease resistance. For example, *SDS2-ACT* showed an increased accumulation of the tyrosinase inhibitors kurarinol and kuraridinol, as well as the phytoalexin (−)-(*S*)-sakuranetin. Interestingly, consistent with transcriptomic results, the levels of carotenoids (dopaxanthin) and several flavonoids are decreased in the mutant. As flavonoids serve as ROS quenchers, lower levels of these compounds are consistent with the increased levels of ROS observed in *SDS2-ACT*.

Consistent with potentially decreased levels of flavonoid data resulting in increased ROS levels, additional transcriptomic and metabolomic data support the role of ROS/redox status in maintaining the *SDS2-ACT* phenotype. Transcriptomic analyses indicated the enrichment of genes involved in the synthesis of glutathione and glutathione conjugates (increased expression of GSTs).

Metabolomic analyses indicated decreased levels of glutathione, which could be explained by the increased conjugation of glutathioine by GTSs’ enzymes, resulting in the depletion of the pool. The activity of GSTs could be serving to protect proteins from the increased ROS present in the *SDS2-ACT* cellular environment, allowed by decreased flavonoid levels. Overall, this could allow these lines to maintain a ROS environment very detrimental to pathogens while at least partially buffering the impact of these ROS on plant proteins.

While the potential impacts of the *SDS2-ACT* activation on terpenoid and phenylpropanoid metabolism are relatively clear, the impacts of this alternation on phytohormone metabolism and physiology requires further investigation. The broad-spectrum metabolomic methods used in this study were not optimal for the extraction and quantification of phytohormones, particularly auxins and related metabolites. However, our study did indicate that the levels of several stress/pathogen-related hormones were increased. Specifically, the levels of bioactive jasmonic acid isoleucine (JA-Ile) and methyl jasmonate, key hormone signals in several pathogen responses, were up-regulated in *SDS2-ACT*, as was the jasmonic acid metabolite 3,6-nondienal. Additionally, the levels of several cytokinins, including N-glycosylzeatin and dihydrozeatin, were also up-regulated in *SDS2-ACT*, although the impact of this up-regulation on disease resistance is unclear. Our transcriptomic, metabolomic, and pathway analyses indicated that the impact of the *SDS2-ACT* mutation on gibberellin physiology is complex, with some GA compounds and precursors showing increased synthesis and accumulation (i.e., GA1, GA3, GA29, etc.), while others showed decreased synthesis and accumulation (GA12, GA4, and GA7). Given the complexity of these interactions, more study is needed to fully understand the potential role of shifts in GA pools on the pathogen-resistance phenotype in *SDS2-ACT* mutants.

Finally, and potentially most interestingly, both transcriptomic and metabolomic data point toward altered levels of ABA and IAA being important in *SDS2-ACT* phenotypes. Both data sets indicate that pathways to degrade ABA and IAA are up-regulated in *SDS2-ACT*. Catabolites or alternate metabolic species for both hormones (8′-hydroxyabscisic acid and phaseic acid in the case of ABA; and phenylacetic acid, or PAA, in the case of auxin) accumulate in *SDS2-ACT*. The accumulation of PAA is particularly interesting. PAA has been proposed to serve as an alternate auxin in most plants (where the “primary” auxin is indole-3-acetic acid [IAA]) [35]. PAA does not appear to be transported directionally through plant tissues in the same manner as auxin and exhibits decreased activity in eliciting auxin responses compared to IAA [35]. However, increased levels of PAA (exogenously applied) have been correlated with pathogen resistance to *Penicillium sclerotinia* and *Penicillium italicum* in citrus [36]. As increased levels of auxin have been linked to increased pathogen sensitivity in soybean (*Phytophthora sojae*) [37], Arabidopsis [38,39,40], and rice [41,42,43], the increasing levels of PAA, potentially at the expense of the levels of free IAA may be contributing to disease resistance in *SDS2-ACT*. Interestingly, auxin accumulations during the early stages of blast infection have been localized to areas immediately adjacent to the hyphae and are thought to be the accumulations of auxins produced by the fungus during the biotrophic stage of the infection [43]. Additionally, one of the functions of the increased PAA levels detected in *SDS2-ACT* compared to Kitaake may be to reduce localized auxin accumulations or to serve as an alternate auxinic compound. In pea (*Pisum sativum*), the exogenous application of PAA blocked the transport of IAA in both intact and excised stem segments [44,45]. It is possible that increased PAA levels in *SDS2-ACT* may be contributing to blast resistance by inhibiting auxin transport and reducing auxin levels in leaves.

## 4. Materials and Methods

### 4.1. Plant Materials and Growth Conditions

Seed sterilization and germination were carried out as previously described [46]. Uniformly germinated seeds of SDS2-ACT and Kitaake were sown in soil pots to ensure even growth conditions, and the seedlings were grown in a growth chamber for 5 weeks. Growth chambers were set to 26 °C during the day and 20 °C at night, with a 12 h/12 h light/dark photoperiod and 80% relative humidity.

### 4.2. RNA Extraction, Library Construction, and RNA-seq

RNA was isolated from the leaf tissues of 5-week-old SDS2-ACT and Kitaake WT rice lines. Leaves were collected at the same time of the day to minimize circadian effects on gene expression. Collected leaves were immediately frozen in liquid nitrogen and stored at −80 °C before further processing. Leaf tissues were ground using a Qiagen TissueLyser (24 Hz, 30 s, 4 cycles, cooling in liquid nitrogen during intervals) to ensure consistent tissue homogenization. For each biological replicate, 200 mg samples were taken for RNA isolation. Total RNA was extracted using TRIzol reagent (ThermoFisher, MA, USA) according to the manufacturer’s protocols and purified using Zymo columns with on-column DNAse treatment (Zymo Research, Irvine, CA, USA). RNA integrity, concentration, and purity were assessed using an Agilent 2100 Bioanalyzer (Agilent Technologies, Santa Clara, CA, USA). Three replicates of RNA extracts were sent to the Campus Chemical Instrument Center (CCIC), at The Ohio State University, for transcriptome library construction and sequencing using the HiSeq 4000 platform.

### 4.3. Transcriptomic Analysis

Raw reads obtained from RNA sequencing were quality-checked using FastQC to ensure high read quality. Cleaned reads were then aligned to the *Oryza sativa* reference genome (IRGSP) using HISAT2 to generate alignment files. Transcriptome assembly analysis identified 55,801 unigenes. After removing the unigenes with no expression, 34,990 unigenes were aligned against the database of Nr (NCBI non-redundant protein sequences), and 28,114 protein-encoding genes were concluded for gene annotation in the Kyoto Encyclopedia of Genes and Genomes (KEGG; http://www.genome.jp/kegg, accessed on 28 February 2024). Differential expression analysis was performed using edgeR in R. Genes exhibiting a log2-fold change ≥ 2 and a *p*-value < 0.05 were classified as DEGs. GO term enrichment analysis was conducted using the agriGO v2.0 (http://systemsbiology.cau.edu.cn/agriGOv2/, accessed on 28 February 2024) classification system [47] to categorize DEGs into biological processes, molecular functions, and cellular components. Significantly enriched categories were identified based on a hypergeometric test with a Benjamini–Hochberg false discovery rate (FDR) correction. The biological processes term was selected with a fold change ≥ 2.0 and significance threshold of *p* < 0.05 to identify overrepresented GO terms among the DEGs. To investigate the functional implications of the DEGs, we performed the Kyoto Encyclopedia of Genes and Genomes (KEGG) pathway enrichment analysis using the R package clusterProfiler (https://github.com/YuLab-SMU/clusterProfiler.git, accessed on 28 February 2024). The results of the KEGG pathway enrichment were visualized using bar charts. To visualize the connection between multiple biological categories and the list of gene information with expression changes, gene-concept network diagrams were generated using the cnetplot function in the R package clusterProfiler in RStudio (R version 4.3.1). The gene-concept network illustrates the relationship between DEGs and enriched pathways, with node size representing the number of genes involved and edge color indicating fold change in gene expression.

### 4.4. Metabolite Extraction and Sample Preparation

For downstream metabolomic analyses, plants were grown as described above. Leaves were harvested at 5 weeks and ground in liquid nitrogen using a Qiagen TissueLyser and metal beads. Ground tissues were transferred into 15 mL conical tubes for storage. To extract metabolites, 200 mg of tissue was transferred to pre-weighed 2 mL microcentrifuge tubes immediately following tissue grinding. Samples were then weighed to 0.1 mg, and extraction solvent (methanol containing indole-3-propionic acid at a concentration of 20 µg/mL as an internal standard) was added to each tube at a ratio of 200 µL per 100 mg tissue. Sample tubes were sealed, and tissues were extracted using gentle nutation at 4 °C for 30 min. Finally, samples were centrifuged at 13,000× *g* for 15 min at 4 °C. Supernatants were harvested and passed through 0.2 µm PVDF disk syringe filters into low-volume inserts seated in amber LC-MS vials. Four biological replicates of each sample were sent to the CCIC for metabolomic analyses, with each biological replicate injected at least three times.

### 4.5. Metabolomic Analyses and Data Processing

Metabolomic analyses were conducted at the CCIC at The Ohio State University. Untargeted analysis was performed on an Agilent 6545 QTOF mass spectrometer with HPLC separation on a Poroshell 120 SB-C18 column (2 × 100 mm, 2.7 µm particle size) using an Infinity 1290 LC system. The gradient consisted of solvent A (H2O with 0.1% formic acid) and solvent B (MeOH with 0.1% formic acid) at a 200 µL/min flow rate, with an initial 2% B, a linear ramp to 90% B at 15 min, up to 95% B for 1 min, and back to 2% B at 17 min, followed by equilibration of 2% B until 30 min. In total, 10 µL was injected for each sample, and the top 5 ions were selected for data-dependent analysis with a 30 s exclusion window. Mass spectra were recorded at a mass range of 50 to 1700 *m*/*z*, using a Dual AJS ESI source with a VCap of 4000 V, a nozzle voltage of 500 V, a nebulizer flow of 8 L/min at 250 °C, and a sheath flow of 10 L/min at 350 °C. To prevent cross-contamination, a blank run was conducted every third injection, and blanks were analyzed for sample carry-over.

For feature selection in the untargeted results analysis, including database comparison and statistical processing, samples were initially analyzed in Progenesis QI. Feature alignment scores were all above 90% after peak picking, and normalization was performed using the internal standard of indole-3-propionic acid. ANOVA *p*-value scores between the SDS2-ACT and Kitaake WT samples were calculated, and features with a *p*-value above 0.05 were removed. Database matching using ChemSpider (version 2.0.2), selecting for adducts M + H, M + Na, M + K, M + 2H, and 2M + H with less than 20 ppm of mass error, was used to tentatively identify potential metabolites. All metabolite identifications are tentative, relying on Progenesis QI software (version 3.0) and the ChemSpider database matching without MS/MS confirmation. To correct for the false discovery rate, only differentially expressed features with *p*-values < 0.05 and q-scores <0.05 (a total of 2348 features) were included in downstream analyses. Principal component analysis (PCA) was conducted using the ‘prcomp’ function in R and visualized using the ggplot2 package. Volcano plots were generated using the ggplot2 package in R, while heatmap plots were generated using the pheatmap package.

Using a Python program developed by co-author Shaoxing Dai, SMILES (Simplified Molecular Input Line Entry System) information for each feature was obtained via putative Chemspider IDs. Additional metabolite information and putative IDs for each feature were acquired from the KEGG and PMN databases using SMILES string searches. Based on these data, pathway enrichment analyses were conducted, and the abundances of specific metabolites between SDS2-ACT and Kitaake were further investigated. Briefly, using *p* < 0.05 as the threshold to screen differentially expressed metabolites, the fold-change (FC) shift in specific metabolites between SDS2-ACT and Kitaake was calculated using the average normalized peak area for each feature. FC > 1 indicated increasing expression in SDS2-ACT (up), while FC < 1 indicated decreasing expression (down). Features were then organized by logFC and sorted in descending order. The top 200 metabolites with the largest increases in SDS2-ACT vs. WT and the top 200 metabolites with the largest decreases in SDS2-ACT vs. WT were selected for further analyses. These top 200 up- and down-regulated metabolites were putatively identified by manually screening the KEGG and PMN database results and selecting the compound IDs with the highest match score and the lowest mass error. The results of these analyses are provided in Supplementary Tables S2–S4.

## 5. Conclusions

1. The *SDS2-ACT* mutant showed alterations to terpenoid metabolism and accumulated sesqui-, di-, and triterpenoids likely to contribute to disease resistance. Additionally, *SDS2-ACT* plants show increased accumulations of sterols which may contribute to disease resistance via either phytohormone signaling (castasterone) or the modulation of membrane physiology (3-hydroxy sterols).
2. *SDS2-ACT* accumulated several secondary metabolites with previously demonstrated antimicrobial activities. These include the flavonols kurarinol, kuraridinol, and sakuranetin; the phenolic compounds 3,4-hydroxybenzoate, coumarin, cinnamyl alcohol, and dihydroconiferol alcohol; the terpenoids oryzalexin A, oryzalexin D, and momilactone A; and several putative alkaloids.
3. Shifts in phenylpropanoid metabolism in *SDS2-ACT* may help maintain a redox environment rich in ROS detrimental to rice pathogens. The increased activity of glutathione transferases and ion transporters in the mutant may help to protect plant proteins and cells from this redox environment.
4. Alterations to the levels of specific phytohormones (cytokinins, GAs, ABA, and auxins) may contribute to disease resistance in *SDS2-ACT*. In particular, increased levels of phenylacetic acid may contribute to this phenotype.
5. The activation of genes encoding PR and resistance-like genes in *SDS2-ACT* may be pivotal for cell death and enhanced resistance in *SDS2-ACT*.

## 6. Limitations of This Study and Future Work

While the combined transcriptomic and metabolomic approach used in this study is useful in providing insights into the metabolic and signaling pathways involved in mediating disease resistance in *SDS2-ACT*, this approach is not without its limitations. For example, while both transcriptomics and the broad-spectrum “pathway-level” metabolomic analyses completed each identified similar metabolic pathways (terpenoid, phenylpropanoid, and flavonoid) as being involved in pathogen resistance, these analyses do not quantify flux through these pathways. Additionally, although the TOF-MS system provides high-resolution mass analysis, definitive structural identification requires MS/MS data. The matching process (Progenesis QI and ChemSpider) offers a standard approach for putative identification but does not eliminate the risk of false positives. Further, as noted above, the conditions used in metabolomic assays were not optimized for the extraction and quantification of phytohormones. Finally, while transcriptomic analyses identified several enzymes involved in signaling, the current study did not quantify the activity of these enzymes, and the current work did not include phosphoproteomics or enzymatic assays to confirm the up-regulation of specific signaling pathways.

Future work will focus on performing targeted liquid chromatography–tandem mass spectroscopy studies combined with NMR to confirm the tentative identities of the molecular species detailed in the current study. Additional studies in which *SDS2-ACT* mutants are fed isotopically labeled precursors (for example, ^13^CO_2_) would allow the quantification of carbon flux through the metabolic pathways highlighted in the current work. Finally, while this study demonstrates correlations between transcriptomic and metabolomic changes in *SDS2-ACT*, particularly in pathways such as diterpenoid and carotenoid biosynthesis, the causal relationships between these molecular changes and the observed resistance phenotype remain to be validated. Future integrative experiments, such as the generation and analysis of null-function mutants for enzymes involved in intermediate metabolite biosynthesis, combined with targeted metabolite quantification, could provide conclusive validation and strengthen our findings.

## Data Availability

Data are contained within the article and Supplementary Materials.

## Supplementary Materials

The following supporting information can be downloaded at: https://www.mdpi.com/article/10.3390/plants14050665/s1, Table S1: Transcriptomic data of *SDS-ACT* and Kitaaki; Table S2: Metabolomic raw data of *SDS-ACT* and Kitaaki; Tables S3–S6: Up- and down-regulated metabolites with ID and annotation. Figure S1: Several Pathogenesis related (PR) genes showed significant up-regulation in the SDS2-ACT mutant. Figure S2. Validation of selected DEGs by qRT-PCR.
