# Supplementary material for: Integrated Transcriptomic and Metabolomic Analyses Reveal the Importance of the Terpenoid, Fatty Acid, and Flavonoid Pathways in Rice Cell Death and Defense
Source: Plants (Basel). 2025 Feb 21;14(5):665. doi: 10.3390/plants14050665 (PMC11901969; doi:10.3390/plants14050665)
Supplement: Supplementary file 1 [file plants-14-00665-s001.zip › Suppl Figures 1 and 2.pptx]

## Slide 1
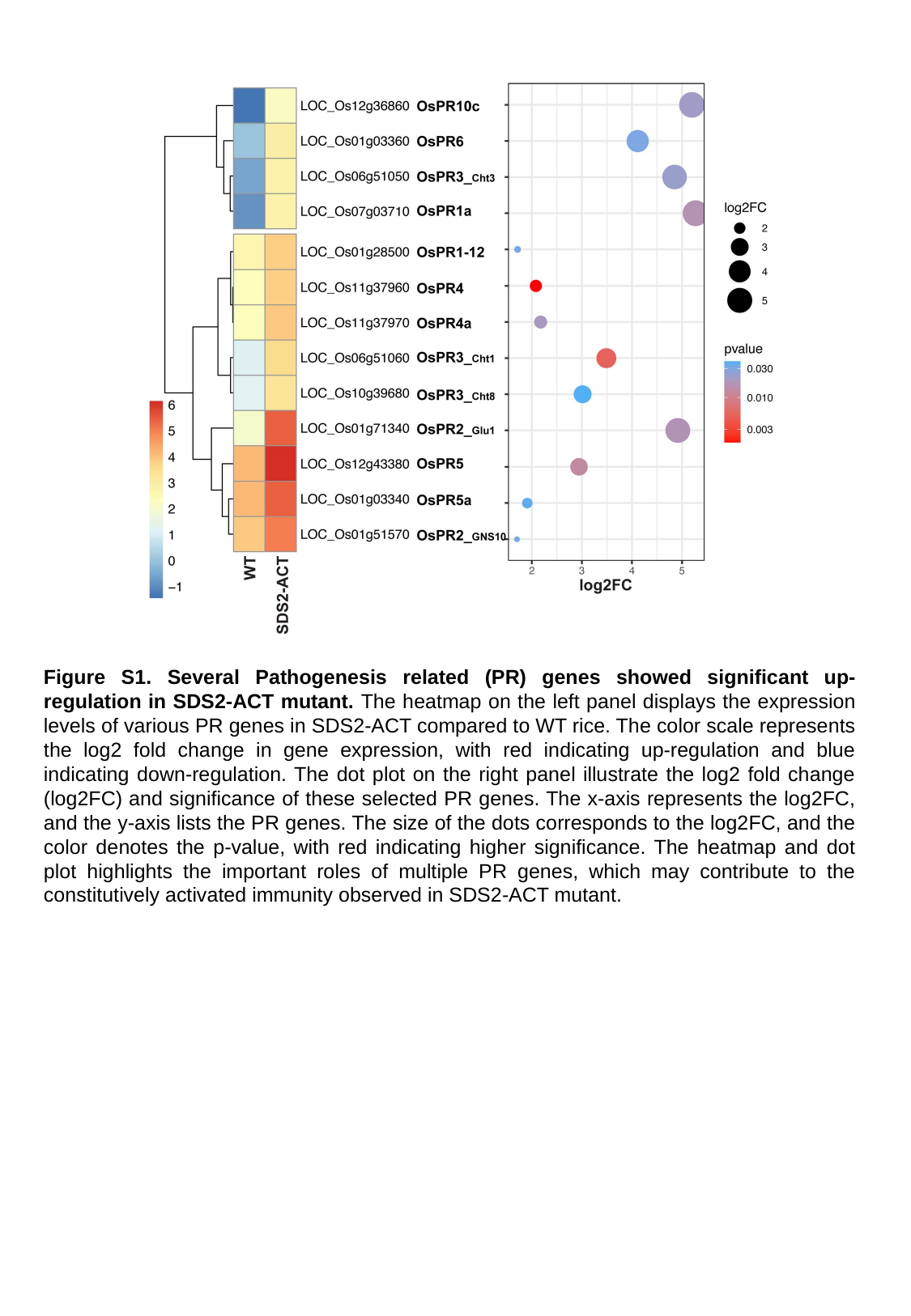

Figure S1. Several Pathogenesis related (PR) genes showed significant up-regulation in SDS2-ACT mutant. The heatmap on the left panel displays the expression levels of various PR genes in SDS2-ACT compared to WT rice. The color scale represents the log2 fold change in gene expression, with red indicating up-regulation and blue indicating down-regulation. The dot plot on the right panel illustrate the log2 fold change (log2FC) and significance of these selected PR genes. The x-axis represents the log2FC, and the y-axis lists the PR genes. The size of the dots corresponds to the log2FC, and the color denotes the p-value, with red indicating higher significance. The heatmap and dot plot highlights the important roles of multiple PR genes, which may contribute to the constitutively activated immunity observed in SDS2-ACT mutant.

## Slide 2
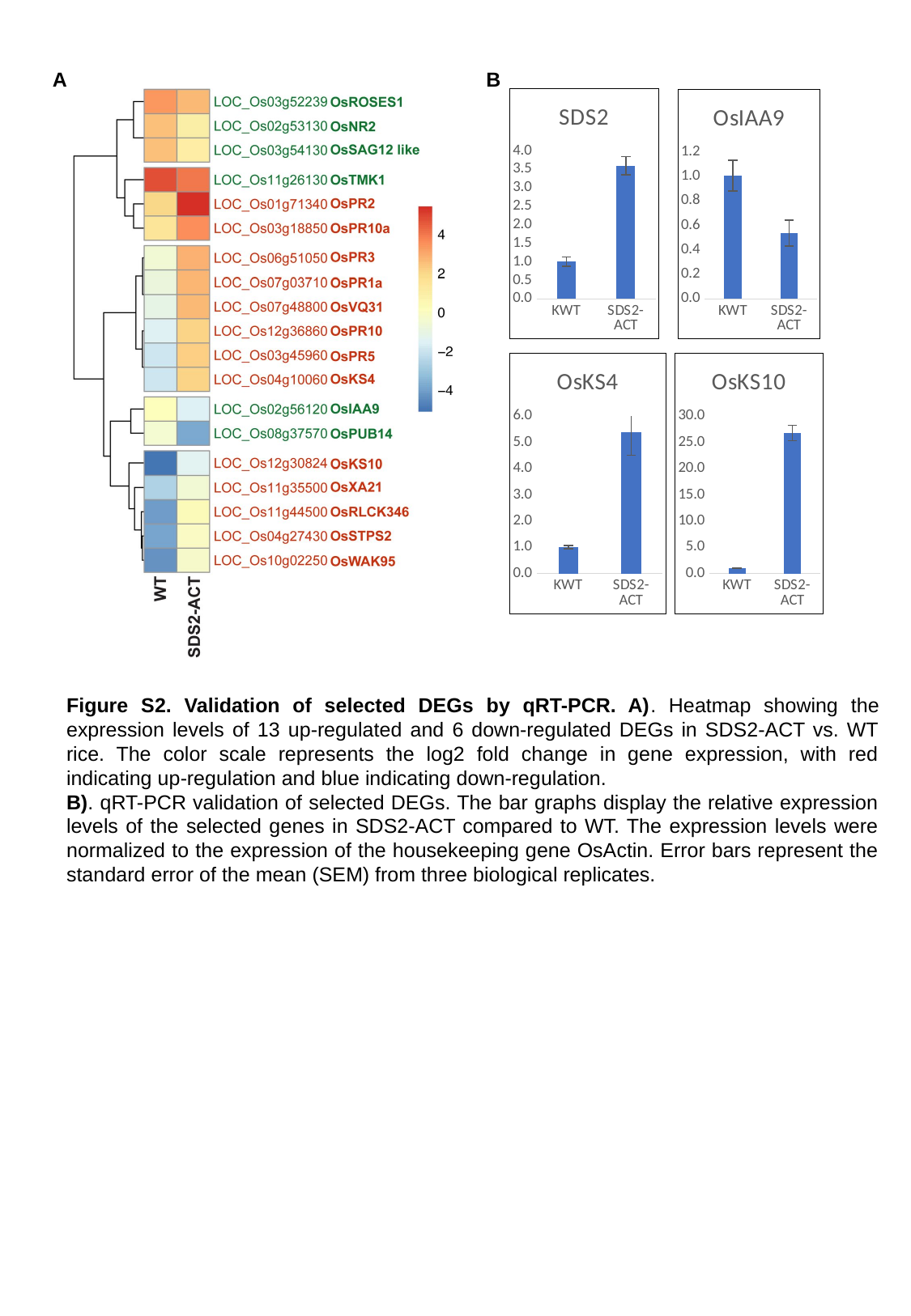

A
B
### Chart:
| Category | SDS2 |
|---|---|
| KWT | 1.0052190324128132 |
| SDS2-ACT | 3.5969887014436197 |
### Chart:
| Category | OsIAA9 |
|---|---|
| KWT | 1.0054860840745288 |
| SDS2-ACT | 0.5367125299753907 |
### Chart:
| Category | OsKS4 |
|---|---|
| KWT | 1.001326033488456 |
| SDS2-ACT | 5.39389419841978 |
### Chart:
| Category | OsKS10 |
|---|---|
| KWT | 1.001587893050948 |
| SDS2-ACT | 26.7792635778718 |Figure S2. Validation of selected DEGs by qRT-PCR. A). Heatmap showing the expression levels of 13 up-regulated and 6 down-regulated DEGs in SDS2-ACT vs. WT rice. The color scale represents the log2 fold change in gene expression, with red indicating up-regulation and blue indicating down-regulation.
B). qRT-PCR validation of selected DEGs. The bar graphs display the relative expression levels of the selected genes in SDS2-ACT compared to WT. The expression levels were normalized to the expression of the housekeeping gene OsActin. Error bars represent the standard error of the mean (SEM) from three biological replicates.
